# Starvation after Cobalt-60 γ-Ray Radiation Enhances Metastasis in U251 Glioma Cells by Regulating the Transcription Factor SP1

**DOI:** 10.3390/ijms17040386

**Published:** 2016-04-05

**Authors:** Tuo Zhao, Hailong Wang, Hong Ma, Hao Wang, Bo Chen, Yulin Deng

**Affiliations:** School of Life Science, Beijing Institute of Technology, Beijing 100081, China; zhaotuobeijing@hotmail.com (T.Z.); whailong1983@126.com (H.W.); 04656@bit.edu.cn (H.M.); wanghao5678@sina.cn (H.W.); h_chenbo@sina.com (B.C.)

**Keywords:** U251 cell line, glioma, radiation, starvation, metastasis

## Abstract

Radiation is of clinical importance during glioma therapy; however, vasculature damage is observed over the treatment course. This type of tissue damage might lead to starvation conditions, affecting tumor metastasis. To test this possibility, we compared starvation conditions in conjunction with radiation treatment to monitor metastatic ability in the U251 glioma cell line. Transcriptome, western blot, and immunofluorescence analyses were used to measure the RNA and protein expression changes of the U251 cells after various treatments. We found that starvation combined with radiation treatment yielded the most significant expression changes in metastasis-related factors compared to that in the control groups. In addition, a metastasis assay was used to directly measure the metastatic ability of the treated cells, which confirmed that the U251 cells treated with starvation combined with radiation possessed the highest metastatic ability. Furthermore, bioinformatics analysis demonstrated that SP1 represented a common transcription factor associated with changes in metastasis-related factors. Blocking SP1 activity by an inhibitor suppressed the starvation-plus-radiation treatment-mediated enhancement of U251 cell metastasis. Our study provides the first evidence that starvation caused by radiation might play a significant role in enhancing the ability of the glioma cell line U251 to metastasize via regulation of the transcription factor SP1.

## 1. Introduction

Radiotherapy represents the first choice in glioma treatment owing to the high risk of surgery and the low permeability of the blood-brain barrier to pharmaceutical agents; however, patients that show post-radiotherapy recurrence usually exhibit low survival rates because the surviving glioma cells acquire increased metastatic ability following radiation treatment [1,2,3]. Numerous factors contribute to tumor metastasis including the cytoskeleton, adhesion molecules, extracellular matrix-associated proteins, and angiogenesis factors [4,5,6,7,8,9,10]. In particular, cytoskeleton-associated proteins such as the main cytoskeleton components actin and tubulin control tumor cell movement during metastasis [4]. Adhesion molecules can either promote or prevent tumor metastasis. Molecules such as integrins enhance tumor metastasis by mediating tumor cell binding with vascular endothelial cells, thus allowing tumor cells to pass through vessel walls to spread toward selected organs [5,7]. Other molecules such as cadherins reduce tumor metastasis by maintaining physical contacts between cells and with the extracellular matrix (ECM), thus preventing tumor cells from detaching from the primary tumor site [8]. Conversely, ECM proteases such as matrix metalloproteinases are tumor metastasis-promoting factors that can degrade the ECM, which functions as a barrier against tumor cells [9]. Whereas such factors have been shown to exhibit biofunctional changes during radiation treatment [11,12,13], the underlying mechanism of radiation-induced metastasis enhancement is still unknown.

Radiotherapy is not a one-time treatment, but rather a prolonged series. This provides time for tumor cells to adapt to the repeating radiation treatments and develop stronger resistance. One aspect of radiation treatment that cannot be ignored is radiation-induced vasculature damage [14,15]. As the main nutrient transport path, damage to the vasculature might cause the tumor cells to become dystrophic and result in their induction under a starvation condition, which has been shown to directly affect the structure, expression, or signaling pathways of metastasis factors [16,17,18]. However, cellular apoptosis/necrosis, an expected outcome under such conditions, is not observed; surviving tumor cells instead show increasing rates of metastasis and recurrence [2,3,19]. Therefore, we hypothesized that radiation-induced cell starvation is a prime factor that might induce tumor metastasis during the course of radiotherapy treatments. Here, we used the U251 cell line and a combined transcriptomics, bio-molecular, and bioinformatics approach to analyze the RNA and protein expression changes in glioma cells after γ-ray radiation.

## 2. Results

### 2.1. Transcriptome Analysis Demonstrated Distinct Expression Profiles Following Radiation and Starvation Treatments

**Figure 1:**
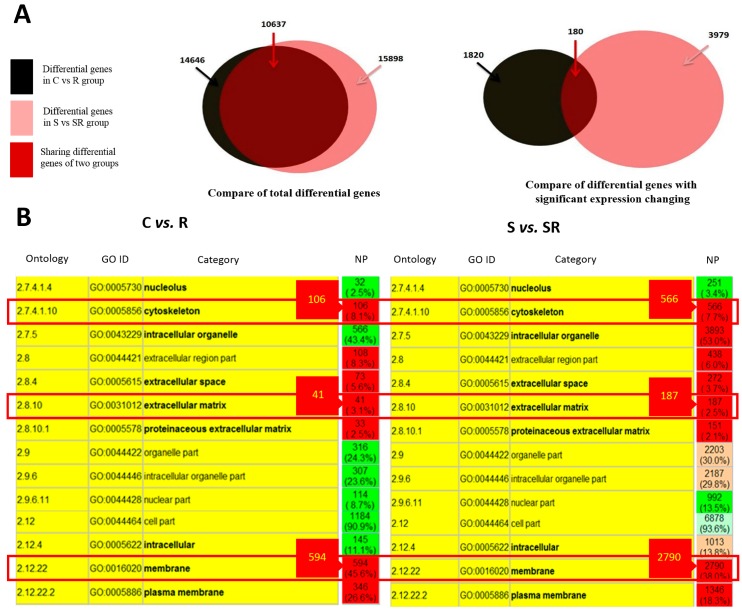
Bioinformatics analysis of differential RNA expression in Control (C) *vs.* radiation (R) groups and starvation (S) *vs.* starvation-radiation (SR) groups. (**A**) Differentially expressed genes in C *vs.* R and S *vs.* SR groups following transcriptome profiling; (**B**) Gene ontology analysis. A larger number of metastasis-related genes were differentially expressed after radiation treatment in the starvation group. NP, numbers of proteins. Red squares indicate the metastasis-related proteins. Red color in NP column indicates the category significant enriched, pink indicates the category enriched, light green indicates the category depleted, green indicates the category significant depleted.

To emphasize that a starvation condition is the essential factor required to induce the metastasis of U251 cells, we categorized U251 cells as control *versus* control-radiation groups and starvation *versus* starvation-radiation groups. First, we analyzed global RNA expression to identify changes in the expression of genes in each of the two groups. Then, we compared the differentially expressed genes and identified that such genes were abundant but dissimilar between the group pairings. The number of genes exhibiting minor expression changes was too high to analyze accurately. Therefore, we compared only the genes that showed significant changes in expression between the two groups in each pair. Compared to the control and starved samples, the irradiated, and the starved plus irradiated groups had 1640 and 3799 differentially expressed genes, respectively. Of these, only 180 genes were common between the control *vs.* radiation and starvation *vs.* starvation-radiation groups. Conversely, genes that had significant expression changes in particular appeared to be unique to each pairing (Figure 1A). Alteration in gene expression can affect various cell characteristics such as metastatic ability. Gene ontology analysis confirmed stronger expression changes in metastasis-related genes after radiation treatment in the starvation group than in the control group. In particular, 566, 187, and 2790 genes associated with the cytoskeleton, ECM, and cell membrane, respectively, exhibited altered expression when cells were starved following radiation. In comparison, only 106 cytoskeletal, 41 ECM, and 594 cell membrane-associated genes showed altered expression upon radiation treatment alone (Figure 1B).

**Figure 2:**
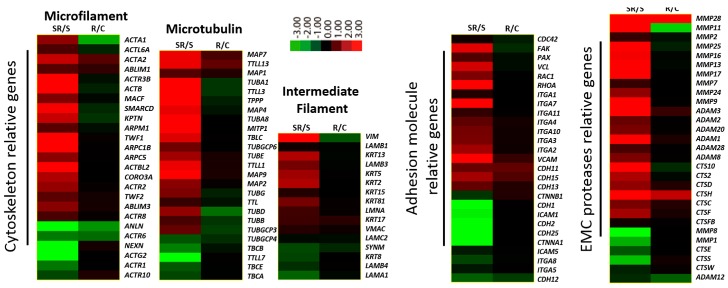
Heat map representations of alterations in expression of metastasis-associated genes in the different treatment groups. The full gene names can be found in Table S1. The fold-changes of SR/S and R/C are illustrated by the color bar, which shows the corresponding log (2) values. C, Control; R, Radiation; S, starvation; SR, starvation + radiation.

**Figure 3:**
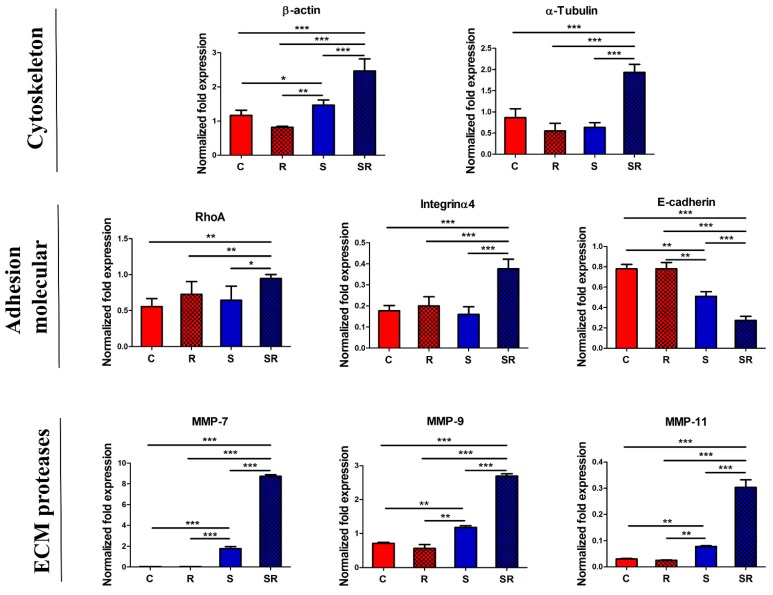
Quantitative real-time PCR validation of the high-throughput sequencing data. The verified genes belonged to the metastasis-related factors including cytoskeleton, adhesion molecule, and ECM proteases. The expression levels of all the selected genes were analyzed by transcriptomics, and confirmed by real-time PCR. Data represent the means ± S.D. of three independent experiments. Expression levels were compared between treated and non-treated groups. C, Control; R, Radiation; S, starvation; SR, starvation + radiation.* *p* < 0.01, ** *p* < 0.001, *** *p* < 0.0001.

To confirm the metastatic advantage conferred to U251 cells upon altered gene expression, we compared the heat maps of the differentially expressed cytoskeletal, adhesion, and ECM protease genes that are strongly associated with metastasis, both within and between groups. The heat maps showed that the expression of most of the cytoskeleton genes (including microfilament-, microtubule-, and intermediate filament-associated genes) increased significantly in the starvation *vs.* starvation-radiation group, which thus might have increased the metastatic ability of the U251 cells. Adhesion-related genes followed a pattern consistent with genes with increased expression being those expected to promote tumor cell metastasis by interacting with vascular endothelial factors. Conversely, genes with decreased expression were those likely to prevent the detachment of tumor cells from the primary tumor site. Genes coding for ECM protease genes had increased expression, which indicated that more ECM proteases could be produced to digest the ECM, thus facilitating the migration of tumor cells. Additionally, genes in all three categories demonstrated more dramatic changes in the starvation *vs.* starvation-radiation group than in control *vs.* control-radiation group, indicating that U251 cells exhibited increased metastatic ability when both starved and irradiated (Figure 2). These findings support that the overall expression of cytoskeletal, adhesion, and ECM protease genes encourage tumor metastasis [4,5,6,7,8,9,10]. Furthermore, we validated the high-throughput RMA sequencing data by confirming the expression of β-*actin* and α-*tubulin* (cytoskeleton), *RhoA*, *integrin* α*4* and *E-cadherin* (adhesion), and *matrix metalloproteinase-7 (MMP-2)*, *MMP-9*, and *MMP-11* (ECM proteases) using real-time PCR (Figure 3).

### 2.2. Starved and Irradiated U251 Cells Display Elevated Protein Expression of Metastatic Factors

**Figure 4:**
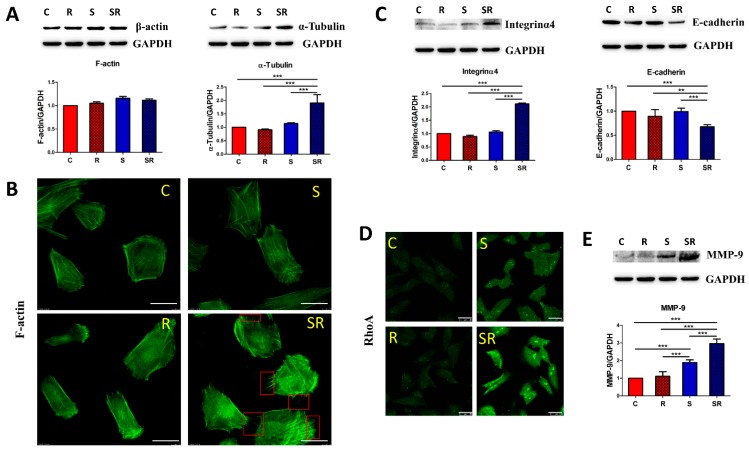
Protein expression of selected metastasis factors in the four different groups. (**A**) Western blot shows the F-actin and α-tubulin expression changes among the four groups; (**B**) F-actin immunofluorescence staining and filopodia counts. The red squares indicate the filopodia on irradiated-starved U251 cells. The scale bar equals 25 μm; (**C**) Integrinα4 and E-cadherin expression was measured by western blot; (**D**) Immunofluorescence detection of RhoA in the four cell groups. The scale bar equals 25 μm; (**E**) Western blot shows MMP-9 expression changes among the four groups. GADPH served as loading control for western blot analyses. The data represent the means ± S.D. of three independent experiments. C, Control; R, Radiation; S, starvation; SR, starvation + radiation. ** *p* < 0.001, *** *p* < 0.0001 *versus* different treated groups.

Based on the RNA expression analysis, we confirmed the protein levels of metastasis-related factors in irradiated starved U251 cells. Among the cytoskeleton molecules, western blot analysis revealed that α-tubulin expression significantly increased in the starvation plus radiation group, whereas F-actin levels remained unchanged (Figure 4A). This result was reflected in the immunofluorescence staining; nevertheless, U251 cells in the starvation-radiation group showed more filopodia, a major motility organelle, than in the other groups (Figure 4B). We next tested proteins associated with adhesion. The starved plus irradiated cells showed significant up-regulation of integrin α4 expression by western blot, which might increase the ability of U251 cells to pass through a vessel wall [20]. Furthermore, the obvious decrease in E-cadherin expression might aid the starved-irradiated U251 cells to detach from the primary tumor site (Figure 4C) [21]. We also detected the expression of RhoA by immunofluorescence. Rho GTPases constitute a family of small signaling G proteins that regulate cytoskeletal arrangement and cell adhesion to mediate tumor metastasis [22]. Notably, there was a dramatic increase in the expression of RhoA, the primary member of the Rho GTPase family [23], in starved plus irradiated cells (Figure 4D). Western blot analysis further indicated that the expression of the metastasis enhancer, MMP9, increased concomitant with the expression of proteins in the ECM protease group (Figure 4E) [24]. These changes in protein levels were consistent with the changes in gene expression, and these alterations might be considered to likely improve the metastatic ability of starved-irradiated U251 cells.

### 2.3. Expression of Metastatic Biomarkers Confirms the Metastatic-Inducing Ability of Starvation-Radiation Combination Treatment in U251 Cells

**Figure 5:**
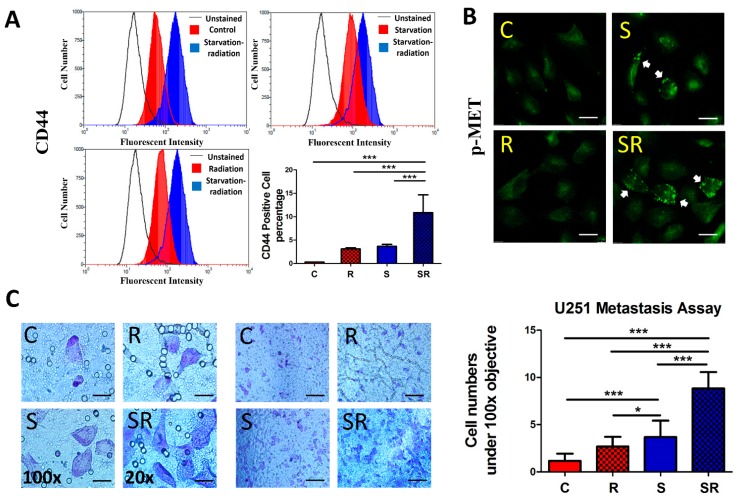
Expression of metastasis biomarkers and metastasis ability in U251 cells were investigated following the four treatments. (**A**) Flow cytometry revealed that the starvation plus radiation group exhibited the highest number of CD44 positive cells; (**B**) Phosphorylated MET signals (white arrow indicating green dots) are expressed in both starved only and starved plus irradiated U251 cells, but the signal is stronger in the latter cells. The scale bar equals 25 μm; (**C**) Following the metastasis assay, U251 cells were observed under 20× (scale bar equals 100 μm) and 100× (scale bar equals 20 μm) magnification, and five random fields were selected to count the metastatic cells. The data represent the means ± S.D. of three independent experiments. Expression levels were compared between treated and non-treated groups. C, Control; R, Radiation; S, starvation; SR, starvation + radiation. * *p* < 0.01, *** *p* < 0.0001 *versus* different treated groups.

To directly assess the effect of the starvation-radiation combination on metastasis, we determined the expression of the metastasis biomarkers CD44 and MET [25,26] in U251 cells. Flow cytometric analysis indicated increased CD44 expression in the starvation plus radiation group (Figure 5A). MET overexpression has been shown to induce tumor growth and angiogenesis, thus facilitating tumor metastasis [27]. Consistent with this, the irradiated-starved U251 cells showed the greatest increase in phosphorylated MET (pMET), which occurred in a punctate pattern (Figure 5B). Furthermore, the metastasis assay indicated that the starved plus irradiated group produced a higher number of cells that passed through the Matrigel and the membrane (Figure 5C).

### 2.4. Starvation-Radiation Treatment Enhances U251 Metastasis by Regulating the Transcription Factor SP1

**Figure 6:**
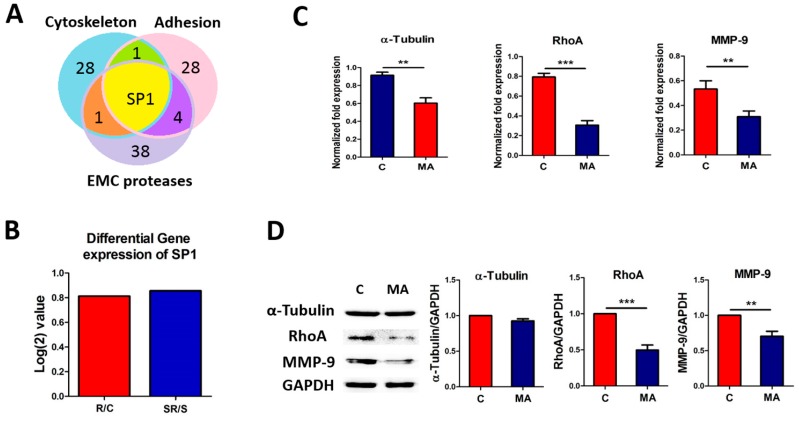
Expression of SP1, the common transcription factor predicted by bioinformatics analysis between differentially expressed metabolism-associated genes, was evaluated following MA inhibitory treatment. (**A**) Three genes were chosen from each metastasis-associated category for use in predicting common transcription factor regulators. The number of transcription factor is shown in the unique or common regions of the Venn diagram; (**B**) The gene expression of *SP1* is upregulated in the radiation (*vs.* control) and starvation-radiation (*vs.* starvation) groups; (**C**) Real-time Q-PCR revealed that the expression of α-Tubulin, RhoA, and MMP-9 at the RNA level were decreased after MA treatment; (**D**) α-tubulin expression showed no significant change by western blot, but RhoA and MMP-9 were down-regulated at the protein level after MA treatment. GADPH served as loading control for western blot analyses. The data represent the means ± S.D. of three independent experiments. C, Control; R, Radiation; S, starvation; SR, starvation + radiation; MA, mithramycin, ** *p* < 0.001, *** *p* < 0.0001 *versus* different treated groups.

**Figure 7:**
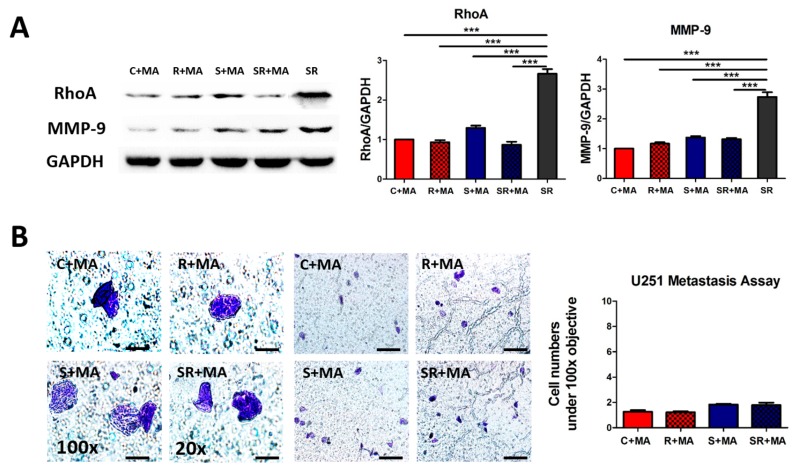
Metastasis factors protein expression and metastasis ability following MA treatment. (**A**) No changes were observed in RhoA and MMP-9 protein expression by western blot. GADPH served as the loading control. The data represent the means ± S.D. of three independent experiments. *** *p* < 0.0001 *versus* different treated groups; (**B**) Metastasis assay is followed the MA treatment, U251 cells were stained by Giemsa dye and the blue-violet cells are observed under 20× (Scale bar equals 100 μm) and 100× (Scale bar equals 20 μm), and five random fields were selected to count the metastatic cells. C, Control; R, Radiation; S, starvation; SR, starvation + radiation; MA, mithramycin.

Bioinformatics analysis indicated that all the metastasis-associated genes could potentially be regulated by a common transcription factor, SP1, despite belonging to different categories (Figure 6A; Figure S1 (supplementary materials)). Additionally, we analyzed SP1 expression using our high-throughput sequencing RNA expression database and found that the gene expression level of SP1 was upregulated both in the radiation (*vs.* control) and in the starvation-radiation (*vs.* starvation) groups (Figure 6B); there was no significant difference in the degree of change in expression level between the two groups. Next, we used the SP1 inhibitor mithramycin (MA) to verify whether SP1 enhances the metastasis ability of U251 cells under starvation-radiation treatment [28,29]. Real-time Q-PCR showed that α-tubulin, RhoA, and MMP-9 transcripts were all downregulated by MA treatment (Figure 6C). Furthermore, western blot results indicated that the changes in RhoA and MMP-9 protein expression were consistent with those at the gene level after MA treatment; however, α-tubulin expression did not vary (Figure 6D). The increased expression of RhoA and MMP-9 disappeared after SP1 inhibition under starvation-radiation conditions, but U251 cells under starvation-radiation condition without MA treatment still present a high expression level of RHOA and MMP-9 (Figure 7A). To confirm the effect of SP1, the metastasis assay was used to directly observe the metastasis ability of MA treated U251 cells under the four various conditions. Distinct from the U251 cells without the MA treatment (Figure 6C), the starvation-radiation condition did not further enhance the metastasis ability upon repression of SP1 activity (Figure 7B).

## 3. Discussion

It has been previously reported that radiotherapy increases the metastasis ability of tumor cells [30,31,32,33]. However, although changes in many signaling pathways have been observed during radiotherapy, there is no recent direct evidence that these biofunctional changes are caused by the radiation. Radiation can induce various stresses in the body, such as the generation of free radicals, hypoxia, hematotoxicity, and starvation [14,34,35,36]; hence, there is an urgent need to clarify whether the enhanced metastasis of tumor cells that manifests during radiotherapy is caused by radiation or by radiation-induced stresses such as starvation conditions. Research has shown that tumors under glucose-starvation conditions utilize stored lactate as an energy source for survival [37]. Additionally, Chen *et al.* demonstrated that the synthesis and uptake of amino acids by cells occurs via common amino acid control pathways during starvation to maintain cell survival [38]. Thus, tumor cells have alternative mechanisms to avoid apoptosis/necrosis during the starvation caused by vascular damage. However, they cannot survive for long under starvation conditions and eventually migrate to a new area where nutrients are available. Therefore, if tumor metastasis is not directly enhanced by radiation, it is possible that radiation-induced starvation might change the metastatic ability of the affected cells. In this study, our results suggested that radiation combined with starvation might enhance the ability of surviving tumor cells to metastasize by regulating the expression of SP1.

No significant change was identified in the expression of metastasis factors between the radiation and control groups, which illustrated that radiation cannot directly induce changes in metastatic ability. Some selected factors in the starvation group did show marked changes in their protein or RNA expression levels; however, the extent of the observed changes was still far less than those seen in the starvation-radiation group, indicating that starvation in conjunction with radiation treatment likely represents the causal condition that enhances metastatic ability. These results verified our hypothesis; however, the complete underlying mechanism is still unknown. We initially found that the changes arose at the gene level and hence we focused on identifying common transcription regulators of the metastasis factors. Notably, bioinformatics analysis identified SP1 as such a common transcription factor. In addition, we demonstrated that the radiation-starvation treatment might occur via SP1-mediated regulation of the expression of metastasis factors at the RNA and protein levels, thus enhancing metastasis.

On the other hand, the degree of change in *SP1* gene expression was the same between the control *vs.* radiation and the starvation *vs.* starvation-radiation groups, indicating that SP1 expression is regulated by radiation but not by starvation. Previous studies have revealed that the DNA binding activity of SP1 can be downregulated by radiation [39] but upregulated by starvation [40]. Taking these findings together, we suggest that in the radiation group, although SP1 expression might be upregulated, a low DNA binding activity limits its effect on the regulation of metastasis-related factors. Conversely, in the starvation-radiation group, radiation increases SP1 expression, and starvation enhances the SP1 binding to the DNA of metastasis-related factors during the process of transcription, thus regulating the expression of such factors. Therefore, the impact of radiation plus starvation on SP1 expression and DNA binding activity appear important to enhance tumor metastasis, and thus the combination of radiation plus starvation might be capable of maximally enhancing the metastatic ability of U251 cells.

## 4. Experimental Section

### 4.1. Cell Line, Cell Culture, and Treatments

The human U251 glioma cell line was purchased from the Cell Center of Peking Union Medical College (Beijing, China) and cultured in minimum essential medium (MEM) supplemented with 10% fetal bovine serum (FBS) (Hyclone, Logan, UT, USA). Cells were seeded in T-25 flasks (Corning, Inc., Corning, NY, USA) with a density of 4 × 10^5^ cells per flask. The culture was maintained at 37 °C in an incubator containing 5% CO_2_.

Cells were separated into four groups as undergoing control, starvation, radiation, and starvation plus radiation conditions. The control and radiation groups were cultured as mentioned above. Cells in the starvation and radiation-starvation groups were starved using MEM without FBS for 12 h prior and subsequent to radiation exposure (evidence supporting the establishment of starvation conditions are shown in Figure S2, supplementary materials). Cells of the radiation and starvation-radiation groups were irradiated using Cobalt-60 (Co-60) γ-rays under atmospheric pressure and ambient temperature (Peking University, Beijing, China). The dose rate was set at 1 Gy/min, the overall treatment time was 7 min, and thus the total dose was 7 Gy. This dose was selected because it represents the half-lethal dose at 12 h, as shown in Figure S3 (supplementary materials). For the treatment with MA, the medium was supplemented with 200 nM MA [41].

### 4.2. High-Throughput Transcriptome Sequencing and Bioinformatics Analysis

Total mRNA from each group was sent to BGI Company and analyzed by high-throughput sequencing (Tables S1 and S2 (supplementary materials), the red color marked genes are selected in heat map illustrations). Gene Ontology enrichment analysis was performed using GOfact Bioinformatics Resources [42], and the results were obtained via “Functional Distribution and Category Enrichment” analysis. The organism chosen was “*Homo sapiens*” because U251 is a human-derived cell line, and the protein identifier type was “HUGO official gene symbol”. The heat map illustrating the dynamic changes of metastasis-associated genes was plotted using Cluster 3.0 (Stanford University, Palo Alto, CA, USA) and Treeview software (Microsoft, Redmond, WA, USA). The impacted transcription factors were predicted using the NCBI database and PROSCAN Version 1.7 [43]. The NCBI gene database was used to determine the location of the target genes on the chromosomes. A 1000-bp nucleotide sequence upstream of the transcriptional “ATG” start site was selected and input into PROSCAN Version 1.7, and the transcription factors of the target genes were automatically predicted after submitting the promoter region. Three significantly changed genes were chosen from the cytoskeleton (F-actin, α-tubulin, and vimentin), adhesion molecule (RhoA, integrinα4, and E-cadherin), and ECM protease (MMP-9, MMP-11, and MMP-25) categories respectively.

### 4.3. Quantitative Real-time Reverse Transcription-PCR (Q-RT-PCR)

Total RNA from the cells in each group was isolated using TRIzol reagent (Invitrogen, Carlsbad, CA, USA). RNA integrity was detected by analyzing 18S and 28S bands on a 1% agarose gel. The purity of all RNA samples was determined by the OD_260_/OD_280_ ratio using a spectrophotometer (Eppendorf, Hamburg, Germany). To detect gene expression by Q-RT-PCR, 2 μg RNA from each sample was reverse transcribed to cDNA. The relative quantitative PCR reaction was set up in a total volume of 15 μL containing 3 μL diluted cDNA, 7.5 μL 2× SYBR green real-time PCR master mix (Toyobo Co. Ltd., Osaka, Japan), 0.5 μL each primer (10 mM), and 4 μL ddH_2_O. Real time Q-PCR was performed on a Bio-Rad IQ^™^5 instrument (Bio-Rad Laboratories, Berkeley, CA, USA). *GAPDH* levels were used for normalization. Primer pairs used for gene amplification are detailed in Table 1.

### 4.4. Western Blot Analysis

Cells were washed twice in cold PBS after harvest, and lysed in ice-cold lysis buffer containing 150 mM NaCl, 1.0% Nonidet-P40, and 50 mM Tris–HCl (pH 8). For western blot analysis, 50 μg protein/lane was subjected to SDS-PAGE and electrotransferred onto a 0.22-μm polyvinylidene fluoride membrane (Millipore, Billerica, MA, USA). The membrane was blocked in 1% TBST containing 5% nonfat dry milk, followed by overnight incubation with primary antibodies including F-actin (CWBIC, Beijing, China), α-tubulin (Cell Signaling, Boston, MA, USA), Rac1 (Santa Cruz, Dallas, TX, USA), E-cadherin (Cell Signaling), or integrin α4 (BioLegend, San Diego, CA, USA). All primary antibodies were diluted 1:1000 in 1% TBST containing 5% nonfat dry milk. The blot was then incubated with suitable horseradish peroxidase conjugated secondary antibodies (CWBIC) followed by chemiluminescence detection (Millipore). GAPDH (Sigma, St. Louis, MO, USA) served as a loading control.

### 4.5. Immunofluorescence

Cells were washed twice with PBS in an 8-well chamber slide (Nunc, Wiesbaden, Germany) and fixed with 4% paraformaldehyde followed by another three PBS washes. Rabbit serum (10%) in PBS was used for blocking non-specific antibody binding. Primary antibodies RhoA (Santa Cruz), Paxillin (Thermo Fisher, Waltham, MA, USA), and p-Met (Cell Signaling) were diluted 1:200 in 0.3% PBS-Triton X-100 and incubated overnight. After three PBS washes, the cells were incubated with a suitable FITC conjugated secondary antibody (CWBIC) or FITC conjugated phalloidin (Sigma) for 2 h. Subsequently, the cells were embedded in Prolong Gold Antifade reagent (Thermo Fisher). Image observation and acquisition after immunofluorescence staining was performed using an Olympus 1X71 fluorescent microscope (Tokyo, Japan).

### 4.6. Flow Cytometric Analysis

Cells were collected and washed twice in PBS. Biotinylated CD44 (BioLegend) was diluted 1:200 with PBS and used to resuspend and incubate cells for 30 min. After two PBS washes, the cells were incubated with PE/Cy5 conjugated streptavidin (BioLegend) for 30 min. After two PBS washes, the cells were analyzed by flow cytometry using the FC500 MCL (Beckman Coulter, Brea, CA, USA).

### 4.7. Metastasis Assay

Matrigel (BioLegend) was diluted in ice-cold serum-free Dulbecco’s modified Eagle medium (DMEM) to a final concentration of 2 mg/mL. Matrigel (100 μL) was added into the upper insert of the transwell and incubated at 37 °C for 2 h allowing the liquid Matrigel to solidify. Then, 5 × 10^4^ cells were suspended in 200 μL serum-free MEM and added in the upper insert (upper compartment) of the 6.5 mm (24-well plate) transwell (Corning). In the well (lower compartment), 600 μL 10% FBS MEM was added. The upper insert was positioned such that the bottom of the upper insert was immersed in the media of the well, and the plate was incubated at 37 °C for 12 h. Post-incubation, the upper insert was removed carefully. A cotton swab was used to gently wipe the upper side of the membrane to remove the non-migrated cells. The cells on the lower side of the insert were fixed with 4% paraformaldehyde for 15 min, followed by staining with 10% Giemsa stain (Solarbio, Beijing, China) for 20 min. The stained insert was washed three times using ddH_2_O and excess water was then immediately removed with a cotton swab. Six random fields of view were chosen for counting under a microscope and averaged.

### 4.8. Statistical Analysis

All experiments were performed in at least three trials and each trial was analyzed in triplicate. The results are shown as the mean values ± standard deviation (S.D.), and statistical significance was evaluated by a Student’s *t*-test; *p* values less than 0.05 were considered statistically significant. In the heat map, log (2) values >2 or <−2 were considered as statistically significant.

## 5. Conclusions

Taking our results together, we suggest that SP1 represents a potential target for radiotherapy in the clinic and that inhibition of the increased expression of SP1 might be a valid strategy to inhibit the enhanced metastasis attained during radiotherapy. We note that because the use of radiotherapy alone results in radioresistance, anti-angiogenesis agents are commonly used in combination. However, this combined treatment can create a starvation-radiation condition that appears to facilitate metastasis and hence, the risks of this approach need to be reevaluated.

## Figures and Tables

**Table 1:** Primer pairs for genes used in quantitative real-time PCR.

Name of Gene	Primer Sequence
*GAPDH* (f)	5′ ACCTGCCGCCTGGAGAAACC 3′
*GAPDH* (r)	3′ GACCATGAGGTCCACCACCCTG 5′
α-*tubulin* (f)	5′ CCAAGCTGGAGTTCTCTA 3′
α-*tubulin* (r)	3′ CAGAGTGCTCCAGG 5′
β-*actin* (f)	5′ AGAGCTACGAGCTGCCTGAC 3′
β-*actin* (r)	3′ AGCACTGTGTTGGCGTACAG 5′
*RhoA* (f)	5′ CCATCATCCTGGTTGGGAAT 3′
*RhoA* (r)	3′ CCATGTACCCAAAAGCGC 5′
*E-cadherin* (f)	5′ ACCACCTCCACAGCCACCGT 3′
*E-cadherin* (r)	3′ GCCCACGCCAAAGTCCTCGG 5′
*Integrin*α*4* (f)	5′ CCACCTTGGTCCTCATGTCAT 3′
*Integrin*α*4* (r)	3′ CATGCGCAACATTCTGATCCT 5′
*MMP2* (f)	5′ CGCCGTCTCCCGTCATCAAA 3′
*MMP2* (r)	3′ TGAGGGTGTCCTCAGCACG 5′
*MMP7* (f)	5′ TGGGCTACGTGACCTATGACAT 3′
*MMP7* (r)	3′ GCCCAGCCACCTCCACTCCTC 5′
*MMP9* (f)	5′ GTATGGGACATTCCTCTGATCC 3′
*MMP9* (r)	3′ CCAATGAATGAATGAATGGATG 5′

f: forword primer, r: reverse primer.

## Supplementary Materials

Supplementary Materials are available online at http://www.mdpi.com/1422-0067/17/3/386/s1.
