# Supplementary material for: Starvation after Cobalt-60 γ-Ray Radiation Enhances Metastasis in U251 Glioma Cells by Regulating the Transcription Factor SP1
Source: Int J Mol Sci. 2016 Apr 5;17(4):386. doi: 10.3390/ijms17040386 (PMC4848883; doi:10.3390/ijms17040386)
Supplement: Supplementary file 1 [file ijms-17-00386-s001.pdf]

Supplementary Materials: Starvation Following Cobalt-60 γ-Ray Radiation Enhances Metastasis in U251 Glioma Cells by Regulating the Transcription Factor SP1

Tuo Zhao, Hailong Wang, Hong Ma, Hao Wang, Bo Chen and Yulin Deng

Table S1. Differential expression of genes between the Starvation *vs.* Starvation-radiation group.

Starvation 12 h-*vs.*-Starvation Radiation 12 h. Gene Differential Expression						
Microfilament Associated Genes						
Gene	Starvation-expression	Starvation-radiation-expression	log2 Ratio (fushe/d uizhao)	*p*-Value	Symbol	Full Name
58	7.08	40.45	2.514318437	1.31×10^{-10}	ACTA1	actin, alpha 1
86	0.82	1.43	0.802319332	0.0458538	ACTL6A	actin-like 6A
59	5.45	33.85	2.634827699	5.26×10^{-9}	ACTA2	actin, alpha 2
3983	1.09	2.01	0.882867366	0.0339902	ABLIM1	actin binding LIM protein 1
57,180	2.045	29.83	3.605910323	3.67×10^{-6}	ACTR3B	actin-related protein 3 homolog B
60	2.72	73.15	4.749179308	2.08×10^{-6}	ACTB	actin, beta
23,499	47.68	115.61	1.277810052	3.44×10^{-13}	MACF1	microtubule-actin crosslinking factor 1
6602	0.27	1.15	2.090602549	0.0196052	SMARCD1	SWI/SNF related, matrix associated, actin dependent regulator of chromatin, subfamily d, member 1
11,133	15.53	41.02	1.401269662	6.92×10^{-11}	KPTN	kaptin (actin binding protein)
84,517	1.36	2.58	0.923764414	0.0257022	ARPM1	actin related protein M1
5756	0.01	0.29	4.857980995	0.047459	TWF1	twinfilin, actin-binding protein, homolog 1
10,095	12.81	177.57	3.793045482	0	ARPC1B	actin related protein 2/3 complex, subunit 1B
10,092	1.63	7.46	2.194303666	0.0002032	ARPC5	actin related protein 2/3 complex, subunit 5
345,651	3.81	64.26	4.076058078	1.99×10^{-7}	ACTBL2	actin, beta-like 2
7464	2.18	7.75	1.829868175	0.0006993	CORO3A	coronin, actin binding protein, 3A
10,097	139.78	396.17	1.502961684	9.21×10^{-14}	ACTR2	ARP2 actin-related protein 2 homolog
11,344	12.26	22.95	0.904535175	0.00176	TWF2	twinfilin, actin-binding protein, homolog 2 (Drosophila)
22,885	105.45	275.68	1.386435512	7.42×10^{-13}	ABLIM3	actin binding LIM protein family, member 3
93,973	2.45	4.3	0.811554911	0.1831722	ACTR8	ARP8 actin-related protein 8 homolog
54,443	6.54	0.29	−4.49516583	1.48×10^{-6}	ANLN	anillin, actin binding protein
72	8.72	3.44	−1.34191957	0.0042654	ACTR6	actin, gamma 6
91,624	0.82	0.01	−6.357552005	0.1383766	NEXN	nexilin (F actin binding protein)
10,121	31.06	0.57	−5.7679521	1.47×10^{-30}	ACTG2	actin, gamma 2
10,120	26.7	8.03	−1.733367849	1.05×10^{-9}	ACTR1	ARP1 actin-related protein 1
23,413	30	17	−0.730707048	0.0073836	ACTR10	ARP1 actin-related protein 10

Gene	Starvation-expression	starvation-radiation-expression	log2 Ratio (fushe/duizhao)	*p*-Value	Symbol	Full Name
**Microtubulin Associated Genes**						
9,053	0.27	1.43	2.404983835	0.0109497	MAP7	microtubule-associated protein 7
440,307	19.89	105.28	2.404116213	0.047459	TTLL13	tubulin tyrosine ligase-like family, member 13
10,051	161.3	214.58	0.411769177	8.92×10^{-7}	MAP1	microtubule-associated protein 1
10,376	5.18	147.16	4.828289673	9.71×10^{-9}	TUBA1	tubulin, alpha 1b
26,140	1.09	30.69	4.815368606	5.08×10^{-5}	TTLL3	tubulin tyrosine ligase-like family, member 3
51,673	0.01	0.57	5.832890014	0.231186	TPPP	tubulin polymerization-promoting protein family member
4,134	44.69	228.92	2.356819562	1.21×10^{-13}	MAP4	microtubule-associated protein 4
51,807	0.01	0.29	4.857980995	0.047459	TUBA8	tubulin, alpha 8
374,655	1.36	10.33	2.925161698	3.15×10^{-5}	TUBA3	tubulin, alpha 3
347,733	2.72	13.2	2.278859373	2.36×10^{-6}	TUBB4	tubulin, alpha 4
85,378	8.99	19.51	1.117820757	0.0001724	TUBGCP6	tubulin, gamma complex associated protein 6
51,175	5.18	15.78	1.607073202	8.26×10^{-6}	TUBE	tubulin, epsilon
25,809	0.27	1.43	2.404983835	0.1094974	TTLL1	tubulin tyrosine ligase-like family, member 1
316	3.81	40.45	3.4082768	1.99×10^{-7}	MAP9	microtubule-associated protein 9
4,133	6.81	17.79	1.385339807	2.22×10^{-5}	MAP2	microtubule-associated protein 2
7,283	1.91	4.3	1.170764022	0.071444	TUBG	tubulin, gamma
150,465	37.06	56.51	0.608643308	0.0001356	TTL	tubulin tyrosine ligase
51,174	0.82	2.29	1.481651783	0.1227506	TUBD	tubulin, delta
7,280	1.36	2.29	0.751740947	0.36957	TUBB	tubulin, beta
10,426	5.45	11.19	1.237881901	0.0076014	TUBGCP3	tubulin, gamma complex associated protein 3
27,229	23.16	8.61	−1.427550111	8.01×10^{-7}	TUBGCP4	tubulin, gamma complex associated protein 4
1,155	88.28	30.98	−1.510749485	5.99×10^{-24}	TBCB	tubulin folding cofactor B
79,739	12.26	0.57	−4.42685325	2.56×10^{-11}	TTLL7	tubulin tyrosine ligase-like family, member 7
6,905	19.62	9.47	−1.050888711	0.0003605	TBCE	tubulin folding cofactor E
6,902	141.14	78.6	−0.844525698	1.10×10^{-15}	TBCA	tubulin folding cofactor A
**Intermediate Filament Associated Genes**						
Gene	Starvation-expression	starvation-radiation-expression	log2 Ratio (fushe/duizhao)	*p*-Value	Symbol	Full Name
23,729	11.99	180.73	3.91393244	9.02×10^{-14}	VIM	Viminten
3,912	306.26	341.37	0.156579326	0.0090365	LAMB1	laminin, beta 1
3,860	5.45	26.11	2.260274323	5.26×10^{-9}	KRT13	keratin 13
3,914	108.72	396.17	1.865502275	3.14×10^{-5}	LAMB3	laminin, beta 3
3,887	7.36	31.56	2.100319534	7.09×10^{-11}	KRT8	keratin 8
3,849	5.18	27.54	2.410504556	9.71×10^{-9}	KRT2	keratin 2
3,866	0.57	1.07	0.587294942	0.6753352	KRT15	keratin 15
3,855	9.54	34.71	1.863290194	8.88×10^{-13}	KRT81	keratin 81
4,000	249.58	637.42	1.352741958	5.56×10^{-12}	LMNA	lamin A/C
3,872	18.53	47.33	1.352892041	8.78×10^{-12}	KRT17	keratin 17
40,0673	7.08	12.91	0.866667735	0.0135918	VMAC	Vinmentin assocaite complex 1
3,918	64.3	39.01	−0.720974739	2.43×10^{-6}	LAMC2	laminin, gamma 2
23,336	54.77	14.63	−1.904456111	8.76×10^{-21}	SYNM	synemin
22,798	1.09	0.57	−0.935294311	0.496384	LAMB4	laminin, beta 4
284,217	27.25	6.31	−2.11054432	2.04×10^{-12}	LAMA1	laminin, beta 1

Adhesion Molecular Associated Genes						
Gene	Starvation-expression	starvation-radiation-expression	log2 Ratio (fushe/duizhao)	*p*-Value	Symbol	Full Name
998	304.08	362.03	0.251658322	2.14×10^{-5}	CDC42	cell division cycle 42
2,197	60.22	350.55	2.541305622	7.04×10^{-14}	FAK	Focal adhesion kinase
389,118	30.24	61.39	1.02154553	5.17×10^{-10}	PXN	Paxillin
7,414	46.05	200.81	2.124558053	5.13×10^{-12}	VCL	vinculin
5,879	12.81	30.69	1.26049817	2.02×10^{-7}	RAC1	ras-related C3 botulinum toxin substrate 1
387	60.76	741.84	3.609914285	1.46×10^{-13}	RHOA	ras homolog gene family, member A
3,672	4.63	5.74	0.310038543	0.518594	ITGA1	integrin, alpha 1
3,679	0.01	1.15	6.845490051	0.0548592	ITGA7	integrin, alpha 7
22,801	23.98	35	0.545523263	0.0065563	ITGA11	integrin, alpha 11
3,676	4.36	8.61	0.981685103	0.025739	ITGA4	integrin, alpha 4
8,515	11.72	26.39	1.171018781	5.51×10^{-6}	ITGA10	integrin, alpha 10
3,675	7.63	17.21	1.173492135	0.0002444	ITGA3	integrin, alpha 3
3,673	9.54	28.69	1.588486797	2.22×10^{-9}	ITGA2	integrin, alpha 2
7,412	1.09	14.92	3.774847495	5.08×10^{-5}	VCAM	vascular cell adhesion molecule
1,009	117.98	236.67	1.004334529	7.36×10^{-13}	CDH11	cadherin 11
1,013	7.9	27.54	1.801604001	1.72×10^{-10}	CDH15	cadherin 15
1,012	14.71	47.62	1.394770374	5.51×10^{-14}	CDH13	cadherin 13
784	33.79	14.92	−1.179348815	2.59×10^{-7}	CTNNB1	catenin (cadherin-associated protein), beta 1
100,129,792	0.27	0.01	−4.754887502	0.526074	CDH1	cadherin 1
3,383	9.54	0.86	−3.471580701	8.37×10^{-8}	ICAM1	intercellular adhesion molecule 1
201,973	36.51	1.43	−4.674204617	1.69×10^{-32}	CDH2	cadherin 2
5,829	308.98	16.35	−4.240150916	3.38×10^{-249}	CDH25	cadherin 25
25,788	0.27	0.01	−4.754887502	0.526074	CTNNA1	catenin (cadherin-associated protein), alpha 1
1,002	4.9	3.44	−0.510373184	0.350316	ICAM5	intercellular adhesion molecule 5
134	14.99	10.04	−0.578241114	0.0631664	ITGA8	integrin, alpha 8
8,516	17.71	5.16	−1.779121241	4.54×10^{-7}	ITGA5	integrin, alpha 5
3,678	272.2	77.74	−1.807938054	1.89×10^{-90}	CDH12	cadherin 12

ECM Protease Associated Genes						
Gene+A107 :A107:G12 8	Starvation-expression	starvation-radiation-expression	log2 Ratio (fushe/duizhao)	*p*-Value	Symbol	Full Name
79,148	4.09	78.6	4.264356564	1.10×10^{-7}	MMP28	matrix metallopeptidase 28
4,320	0.01	0.29	4.857980995	0.47459	MMP11	matrix metallopeptidase 11
2,229	2.18	2.87	0.396722602	0.568192	MMP2	matrix metallopeptidase 2
64,386	0.01	0.57	5.832890014	0.231186	MMP25	matrix metallopeptidase 25
4,325	0.54	3.16	2.548893246	0.0100016	MMP16	matrix metallopeptidase 16
4,322	0.54	9.75	4.174370906	0.0001209	MMP13	matrix metallopeptidase 13
4,326	0.01	0.57	5.832890014	0.231186	MMP17	matrix metallopeptidase 17
4,316	1.91	2.58	0.433798427	0.554742	MMP7	matrix metallopeptidase 7
10,893	1.91	4.02	1.073622863	0.105141	MMP24	matrix metallopeptidase 24
4,327	0.54	6.88	3.671377253	0.000124	MMP9	matrix metallopeptidase 9
8,747	0.01	0.29	4.857980995	0.47459	ADAM3	ADAM metallopeptidase domain 3
53,616	6.54	10.61	0.698062115	0.0632056	ADAM2	ADAM metallopeptidase domain 2
8,748	5.99	10.61	0.824796748	0.0320864	ADAM20	ADAM metallopeptidase domain 20

6,868	0.82	12.34	3.911574675	7.93E-05	ADAM1	ADAM metallopeptidase domain 1
5,428	2.72	4.59	0.754887502	0.1969564	ADAM28	ADAM metallopeptidase domain 28
101	3.54	9.18	1.374744793	0.0025995	ADAM8	ADAM metallopeptidase domain 8
3,476	4.36	70.86	4.022571425	6.01×10^{-8}	CTSW	cathepsin W
1,513	1.36	5.45	2.002649578	0.002743	CTSK	cathepsin K
1,509	1,259.08	3,040.8	1.272080978	1.54×10^{-11}	CTSD	cathepsin D
1,512	1.36	18.36	3.754887502	3.13×10^{-5}	CTSH	cathepsin H
1,075	6.54	15.49	1.243974603	0.0025599	CTSC	cathepsin C
8,722	90.46	300.06	1.72989911	0.0002713	CTSF	cathepsin F
79,000	2.45	2.87	0.228268988	0.734438	CTSA	cathepsin A
4,317	0.27	0.01	−4.754887502	0.526074	MMP8	matrix metallopeptidase 8
4,312	1.36	0.29	−2.229481846	0.142751	MMP1	matrix metallopeptidase 1
1,510	11.44	4.3	−1.411678487	0.0006346	CTSE	cathepsin E
1,520	4.36	0.86	−2.34191957	0.0037043	CTSS	cathepsin S
54,108	6.27	4.59	−0.449971289	0.345238	CTSB	cathepsin B
8,038	21.25	16.64	−0.352807408	0.1587036	ADAM12	ADAM metallopeptidase domain 12

Table S2. Differential expression of genes between the Control *vs.* Radiation group.

Control 12 h-*vs.*-Radiation 12 h. Gene Differential Expression						
**Microfilament Associated Genes**						
Gene ID	C12h-Expression	R12h-Expression	log2 Ratio (R12h/C12h)	Up-Down-Regulation (R12h/C12h)	*p*-value	Symbol
58	4	1	−1.91128	Down	0.248864	ACTA1
86	478	342	−0.39429	Down	0.00010528	ACTL6A
9,185	14	22	0.740797	Up	0.1323372	ACTA2
3,983	28	41	0.638918	Up	0.0696848	ABLIM1
57,180	53	44	−0.17977	Down	0.545756	ACTR3B
60	67,835	49,572	−0.36378	Down	0	ACTB
23,499	2443	2,160	−0.0889	Down	0.0369138	MACF1
6,602	78	32	−1.19668	Down	0.00141277	SMARCD1
11,133	112	37	−1.50918	Down	0.342884	KPTN
84,517	24	18	−0.32632	Down	0.481136	ARPM1
5,756	1,196	1,308	0.217866	Up	0.00015875	TWF1
10,095	1,592	1,484	−0.01263	Down	0.808922	ARPC1B
10,092	1,792	1,501	−0.16693	Down	0.0009321	ARPC5
2,720	33	33	0.088721	Up	9.88×10^{-7}	ACTBL2
57,175	307	310	0.10275	Up	0.37599	CORO3A
10,097	2,478	2,756	0.24212	Up	1.29×10^{-9}	ACTR2
11,344	174	194	0.24569	Up	0.1026536	TWF2
22,885	1,612	1,986	0.389735	Up	2.48×10^{-12}	ABLIM3
93,973	216	181	−0.16632	Down	0.253484	ACTR8
54,443	10	2	−2.23321	Down	1.54×10^{-155}	ANLN
653,857	64	20	−1.58935	Down	0.53121	ACTR6
91,624	470	589	0.414328	Up	3.18×10^{-6}	NEXN
10,096	2,651	2,701	0.115678	Up	0.00335676	ACTG2
10,121	604	609	0.100614	Up	0.224372	ACTR1
10,120	16	23	0.612283	Up	0.222624	ACTR10
**Microtubulin Associated Genes**						
Gene ID	C12h-Expression	R12h-Expression	log2 Ratio (R12h/C12h)	Up-Down-Regulation (R12h/C12h)	*p*-value	Symbol
9,053	3	5	0.825686	Up	0.449272	MAP7
440,307	1	2	1.088721	Up	0.580018	TTLL13
4,130	2,257	2,991	0.494942	Up	0	MAP1
10,376	4,830	3076	−0.56225	Down	1.88×10^{-65}	TUBA1
7,316	55	36	−0.52271	Down	0.997364	TTLL3
11,076	8	6	−0.32632	Down	0.6942	TPPP
4,134	2,771	2,256	−0.20792	Down	3.55×10^{-7}	MAP4
51,807	3	3	0.088721	Up	0.932904	TUBA8

10,381	315	304	0.03744	Up	0.745866	TUBB3
5,066	314	267	−0.15436	Down	1.24×10^{-7}	TUBB4
85,378	95	96	0.103828	Up	0.617974	TUBGCP6
51,175	390	632	0.785171	Up	0.0903884	TUBE
25,809	47	79	0.782059	Up	0.54082	TTLL1
79,884	476	766	0.766793	Up	7.71×10^{-6}	MAP9
4,133	140	152	0.207365	Up	0.219574	MAP2
7,283	319	138	−1.12017	Down	0.033937	TUBG
23,168	55	32	−0.69264	Down	0.705502	TTL
8,626	877	196	−2.073	Down	0.53121	TUBD
81,027	15	6	−1.23321	Down	0.6942	TUBB
10,426	8	3	−1.32632	Down	2.66×10^{-9}	TUBGCP3
27,229	422	196	−1.01767	Down	0.00585406	TUBGCP4
1,155	590	314	−0.81817	Down	0.210634	TBCB
79,739	132	140	0.17361	Up	0.320934	TTLL7
6,905	149	142	0.019299	Up	0.90747	TBCE
6,902	641	619	0.038336	Up	0.636618	TBCA

Intermediate Filament Associated Genes

Gene ID	C12h-Expression	R12h-Expression	log2 Ratio (R12h/C12h)	Up-Down-Regulation (R12h/C12h)	*p*-value	Symbol
7,431	1,190	469	−1.25458	Down	8.28×10^{-14}	VIM
3,912	1,429	1,556	0.211557	Up	0.00035589	LAMB1
164	6,887	6,082	−0.09061	Down	6.21×10^{-5}	KRT13
23,764	85	90	0.171183	Up	0.432332	LAMB3
283,987	1	1	0.088721	Up	0.954202	KRT8
7,408	416	447	0.192412	Up	0.050155	KRT2
3,866	12	14	0.311113	Up	0.585734	KRT15
5,881	5	5	0.088721	Up	0.916952	KRT81
54,872	237	263	0.238896	Up	0.0642952	LMNA
255,928	285	538	1.005365	Up	5.73×10^{-13}	KRT17
400,673	34	45	0.493111	Up	0.585734	VMAC
1,289	84	42	−0.91128	Down	0.662324	LAMC2
23,336	12	4	−1.49624	Down	0.0242432	SYNM
3,856	122	144	0.327908	Up	0.0643998	KRT8
22,798	16	17	0.176184	Up	0.725082	LAMB4
64,427	49	33	−0.4816	Down	0.595896	LAMA1

Adhesion Molecular Associated Genes

Gene ID	C12h-Expression	R12h-Expression	log2 Ratio (R12h/C12h)	Up-Down-Regulation (R12h/C12h)	*p*-value	Symbol
998	132	89	−0.47994	Down	0.661508	CDC42
8,263	598	316	−0.8315	Down	0.555232	FAK
4,942	339	245	−0.37978	Down	0.673	PXN
7,414	168	144	−0.37071	Down	9.68×10^{-6}	VCL
6,996	404	394	0.052561	Up	0.606154	RAC1
387	3,339	3,289	0.066954	Up	0.0588632	RHOA
3,672	300	285	0.01472	Up	0.90059	ITGA1
8,029	15	15	0.088721	Up	0.862988	ITGA7
22,801	994	1,022	0.128798	Up	0.0450204	ITGA11
3,676	21	25	0.340259	Up	0.01785044	ITGA4
8,515	150	183	0.375602	Up	0.426862	ITGA10
3,675	3,068	3,446	0.256345	Up	0.00171727	ITGA3
93,953	5	6	0.351755	Up	0.692642	ITGA2
7,412	77	119	0.716752	Up	0.730576	VCAM
1,009	708	1,433	1.108086	Up	8.43×10^{-5}	CDH11
1,013	47	90	1.025985	Up	0.0368506	CDH15
1,012	614	780	0.433956	Up	2.18×10^{-8}	CDH13
56,998	17	20	0.323186	Up	0.498704	CTNNB1
1,016	1	1	0.088721	Up	0.954202	CDH1
3,385	56	59	0.164009	Up	0.541718	ICAM1
1,000	1,832	1,680	−0.03624	Down	0.457552	CDH2
1,002	60	57	0.01472	Up	0.95322	CDH25
1,495	3,175	3,281	0.1361	Up	0.00015071	CTNNA1
3,383	225	233	0.139126	Up	0.301872	ICAM5
3,678	2,658	2,592	0.052445	Up	0.1877826	ITGA8

Gene ID	C12h-Expression	R12h-Expression	log2 Ratio (R12h/C12h)	Up-Down-Regulation (R12h/C12h)	*p*-value	Symbol
84,107	13	13	0.088721	Up	0.723518	ITGA5
1,010	86	34	−1.25008	Up	0.8719	CDH12
**ECM protease Associated Genes**						
Gene ID	C12h-Expression	R12h-Expression	log2 Ratio (R12h/C12h)	Up-Down-Regulation (R12h/C12h)	*p*-value	Symbol
79,148	1	6	1.422698	Up	0.0584136	MMP28
4,320	11	2	−2.37071	Down	0.01764836	MMP11
4,313	7,722	7,988	0.13758	Up	2.30×10^{-9}	MMP2
64,386	4	3	−0.32632	Down	0.79472	MMP25
4,325	78	71	−0.04693	Down	0.845974	MMP16
4,322	8	7	−0.10392	Down	0.901464	MMP13
2,029	860	816	0.012953	Up	0.853502	MMP17
4,316	1	1	0.088721	Up	0.954202	MMP7
10,893	113	116	0.126523	Up	0.506342	MMP24
26,973	256	249	0.048723	Up	0.70337	MMP9
203,102	2	3	0.673683	Up	0.630906	ADAM3
8,745	3	3	0.134834	Up	0.0978672	ADAM2
253,430	51	57	0.249185	Up	0.370204	ADAM20
8,759	24	32	0.503758	Up	0.1961388	ADAM1
10,863	99	97	0.059277	Up	0.553324	ADAM28
89,953	33	37	0.25378	Up	0.462814	ADAM8
1,521	13	6	−1.02676	Down	0.1507034	CTSW
1,513	21	17	−0.21613	Down	0.723518	CTSK
1,509	17	23	0.52482	Up	3.37×10^{-6}	CTSD
8,722	2	6	1.673683	Up	0.256566	CTSH
1,519	150	194	0.459815	Up	0.00325642	CTSC
10,423	110	146	0.497186	Up	0.00611926	CTSF
5,476	1,084	1,124	0.140998	Up	0.0216588	CTSA
4,323	105	109	0.034782	Up	0.858242	MMP8
4,312	2	2	0.088721	Up	0.94252	MMP1
1,510	2	2	0.088721	Up	0.94252	CTSE
1,520	391	436	0.24588	Up	0.01432658	CTSS
25,994	741	767	0.138474	Up	0.0623412	CTSB
51,512	284	93	−1.52187	Down	2.82×10^{-21}	ADAM12

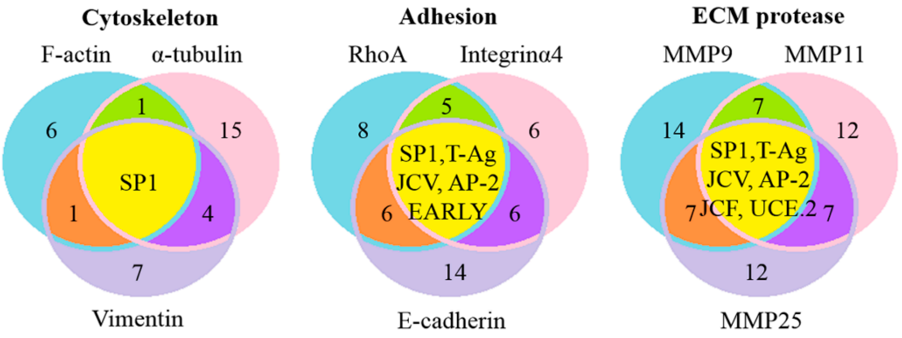

Figure S1. Common transcription factors of genes in the three metastasis related categories.

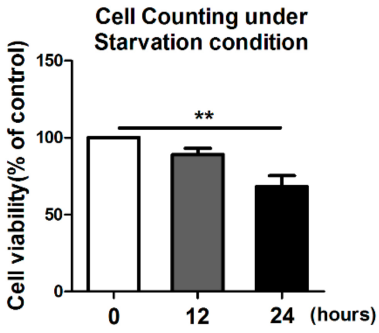

Figure S2. Proof of starvation conditions at 12 and 24 h. ** $p < 0.001$ *versus* different treated groups.

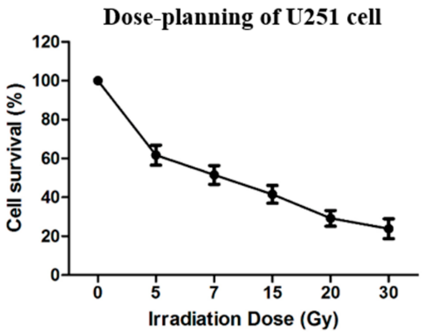

Figure S3. Dose-response of U251 cells.
